# The effect of the first vaginal birth on pelvic floor anatomy and dysfunction

**DOI:** 10.1007/s00192-019-04044-2

**Published:** 2019-07-20

**Authors:** Iva Urbankova, Klara Grohregin, Jiri Hanacek, Michal Krcmar, Jaroslav Feyereisl, Jan Deprest, Ladislav Krofta

**Affiliations:** 1 Institute for the Care of Mother and Child and Third Faculty of Medicine, Charles University, Podolske nabrezi 157, 14700 Prague, Czech Republic; 2 Department of Development and Regeneration, Organ systems cluster, Group Biomedical Sciences, and Pelvic Floor Unit, University Hospitals KU Leuven, Leuven, Belgium

**Keywords:** BMI, Forceps, Incontinence, Maternal age, Pelvic organ prolapse

## Abstract

**Introduction and hypothesis:**

First vaginal delivery severely interferes with pelvic floor anatomy and function. This study determines maternal and pregnancy-related risk factors for pelvic floor dysfunction (PFD), including urinary incontinence (UI), urgency, anal incontinence (AI), pelvic organ prolapse (POP) and levator ani muscle (LAM) avulsion.

**Methods:**

This is a single-centre prospective observational cohort study on healthy women in their first singleton pregnancy. All underwent clinical and 3D transperineal ultrasound examination at 6 weeks and 12 months postpartum. Objective outcomes were POP-Q and presence or absence of LAM trauma. Functional outcomes were measured by the ICIQ-SF and PISQ 12. Multivariate regression was performed to determine birth and maternal habitus-related risk factors for UI, urgency, AI, dyspareunia, LAM avulsion and ballooning.

**Results:**

Nine hundred eighty-seven women were included. Risk factors for UI were maternal age per year of age (OR: 1.09; 95% CI: 1.04–1.13; *p* = 0.0001) and BMI before pregnancy (OR: 1.08; 95% CI: 1.04–1.13; *p* = 0.001); for POP stage II+ maternal age (OR: 1.08; 95% CI: 1.08–1.14; *p* = 0.005). Avulsion was more likely after forceps (OR: 3.22; 95% CI:1.54–8.22; *p* = 0.015) but less likely after epidural analgesia (OR: 0.58; 95% CI: 0.37–0.90; *p* = 0.015) and grade I perineal rupture (OR: 0.50; 95% CI: 0.29–0.85; *p* = 0.012). Ballooning was more likely at increased maternal age (OR: 1.08; 95% CI: 1.02–1.13; *p* = 0.005), epidural (OR: 1.64; 95% CI: 1.06–2.55; *p* = 0.027) and grade I perineal rupture (OR: 1.79; 95% CI: 1.10–2.90; *p* = 0.018).

**Conclusion:**

Though maternal characteristics at birth such as age or BMI increase the risk of PFD, labour and birth factors play a similarly important role. The most critical risk factor for MLA avulsion was forceps delivery, while an epidural had a protective effect.

**Electronic supplementary material:**

The online version of this article (10.1007/s00192-019-04044-2) contains supplementary material, which is available to authorized users.

## Introduction

Pelvic floor dysfunction (PFD), such as pelvic organ prolapse (POP), urinary (UI) and anal incontinence (AI), affects many women worldwide with millions of them undergoing corrective surgery at significant expense and personal suffering [1]. Many risk factors for development and symptom progression were identified, and many of them are shared by different PFDs [2]. According to DeLancey’s lifespan model, PFD becomes symptomatic when the pelvic floor function drops under a certain threshold level [3]. Following an initial drop caused by pregnancy and birth, other risk factors such as lifestyle, smoking, being overweight and chronically increased intra-abdominal pressure negatively affect its function. In most women, PFD becomes symptomatic after several decades, but women with severe pelvic floor trauma may become symptomatic shortly after their first birth.

The most frequently shared risk factor for all PFDs is vaginal birth. Apart from the effects of pregnancy, vaginal birth additionally interferes directly with all structures and tissues of the pelvic floor [4]. The detrimental nature of the impact of vaginal delivery is even more pronounced in the case of forceps extraction to complete the second stage of labour [5]. Other obstetric risk factors include high foetal birth weight, a prolonged second stage of labour and high BMI [6]. Some studies had also described high maternal age at the time of first birth, but that is controversial [7, 8].

To contribute to the study of the effects of vaginal birth, we set up a large prospective cohort study of unselected nulliparous women, which we followed from birth to 1 year postpartum. Herein we correlate the demographic and obstetric risk factors for the presence of PFD 1 year after the birth. An additional goal was to identify the risk factors for levator ani muscle trauma, as diagnosed by ultrasound, and, if applicable, its contribution to the presence of PFD.

## Materials and methods

This single-centre longitudinal study was designed to recruit a large prospective cohort of healthy women in their first pregnancy, including singleton ones only, and who delivered vaginally at or beyond 37 weeks. All women admitted to the labour suite between May 2011 and July 2013 were invited to participate. Exclusion criteria for entry were being minors, not speaking fluent Czech, being non-Caucasian and post-hoc women who became pregnant during follow-up. Women who delivered with unscheduled caesarean section will be reported separately. The institutional ethics committee approved this study, and all women gave written informed consent.

### Follow-up and outcome measures

Before discharge from the birth unit, we asked consenting women about the presence of involuntary leakage of urine or stools before and during pregnancy. Study visits were arranged 6 weeks and 1 year after birth. At these, they were asked about PFD, and they were assessed by one of four experienced nurses from the urogynaecological clinic. Women filled out two validated questionnaires, i.e., the short form of the International Consultation on Incontinence Questionnaire (ICIQ-SF) and Pelvic Organ Prolapse/Urinary Incontinence Sexual Questionnaire (PISQ12) (Czech version) [9, 10]. Women were also explicitly asked if they had any anal and urinary incontinence and dyspareunia. The anatomical assessment was by the pelvic organ prolapse score (POP-Q) [11] and stage and pelvic floor muscle strength assessment by the Oxford scale [12]. Herein clinically significant POP will be defined as the occurrence of stage II+ prolapse (leading point of the prolapse at least at POP-Q point 0 or further) [13]. Transperineal pelvic floor ultrasound (TPUS) was performed as described by Dietz et al. [14] (4.0–9.0-MHz probe, Voluson Expert E8, General Electric, Zipf, Austria). Briefly, the probe was placed vertically over the perineum. A two 4D loop including “squeeze-relaxation-Valsalva manoeuvre-relaxation” was recorded and stored for offline assessment. The nurses had ≥ 3 years of experience with TPUS and clinical evaluation of PFD. Three other similarly qualified observers not involved in the earlier clinical or ultrasound assessment read the TPUS volumes offline (4DView, GE). All observers evaluated the first 55 cases, and their intraclass correlation and kappa coefficient were calculated to confirm their agreement (supplementary Table 1). Later, each case was evaluated by one observer.

The better loop was used for the evaluation and measurement of the genital hiatus at minimum hiatal diameter during contraction and Valsalva and at rest. The levator ani avulsion was identified in tomographical sections centred around the level of minimal hiatal dimension. A set of eight slices was obtained in the axial plane at 2.5-mm intervals from 5 mm below to 12.5 mm above the plane of minimal hiatal dimension. The LAM avulsion was present if there was a discontinuity of the LAM on the three central slices at the muscle contraction [14]. Ballooning was defined as a genital hiatus area at Valsalva ˃ 25 cm^2^ [14]. In case of poor image quality, the US was reviewed and evaluated in cooperation with the supervisor (LK).

During postnatal consultations, we recommended all women undergo pelvic floor training (PFT), which is covered by the national health insurance.

Additional demographic, biometric and obstetric data were obtained from the medical records, including onset of labour, use of oxytocin during labour, epidural or other analgesics, length of the first and second stage, spontaneous or instrumental vaginal birth, or, if appropriate, the use of caesarean section, cervical dilation at that moment and the leading indication, and, if applicable, any trauma to the vagina, vulva or anal sphincter. Perineal trauma was categorized as grade I (perineal skin/vaginal mucosa), grade II (fascia, muscles, perineal body) or grade III (grade II + anal sphincter; irrespective of episiotomy) [15].

### Birth management

At our institution, midwives primarily manage most of the first and second stage, but under the supervision of a gynaecologist. We adhere to the principles of “active management of labour” [16]. For pain relief in the first labour stage, either nalbuphine (10 mg/3 h, i.v.) or “delayed walking” epidural analgesia (EDA) consisting of bupivacaine 0.5% and sufentanil (i.e., cervical dilatation ≥ 4 cm) was offered [17]. Following 30 min in a supine position, the parturient was advised to move and walk actively. If needed, EDA was reloaded every 2 h. Following an uncomplicated first stage, active management of the second stage included encouraging pushing the head down once at stage +3. Midwives recognized the second stage during a vaginal examination and recorded its length. During crowning, perineal protection included manual support and controlled foetal head passage [18]. Left mediolateral episiotomy was performed when it was clinically indicated (i.e., foetal distress, rigid perineum during crowning), but there was no formal policy.

### Statistical evaluation

Data were stored in a purpose-designed database (Office Excel 2007, Microsoft Corp., Redmond, WA, USA) and analysed with SPSS 19.0 (SPSS Inc., Chicago, IL). Only data from women who delivered vaginally were used to determine maternal and obstetrical risk factors for LAM avulsion and PFD 12 months postpartum. Univariate analysis was performed on maternal (age, BMI before pregnancy and at delivery from which the BMI change was calculated) and obstetrical (foetal weight, length of 1st and 2nd stage, type of analgesia, perineal injury, forceps birth, breastfeeding) characteristics. Variables with *p* ˂ 0.250 were taken into account for multivariate regression analysis, using a forward elimination of covariates according to the lack of significance. The risk for symptomatic stress UI and POP in women with LAM avulsion was tested using a chi-square test.

## Results

The study included 3648 women, of whom 1359 completed all study visits (drop-out rate: 62.8%). Of these, we excluded 24 who became pregnant again within 1 year. Three hundred forty-eight (18.6%) women who had a caesarean section were not included in this analysis. Finally, we evaluated 987/3648 (27.0%) women who delivered vaginally. Tables 1 and 2 display comparison of the demographic and obstetric characteristics of included and excluded women. Included women were older (+0.8 year) and were more likely to have had labour induced (+6%).

**Table 1:** Demographic and obstetric characteristics of non-responders and responders

	Non-responders	Responders	*p*	Study group
	*n* = 2313	*n* = 1335		*n* = 987
Demographics				
Age (mean ± SD; years)	30.0 ± 4.0	30.8 ± 3.5	0.0001	30.5 ± 3.4
Height (mean ± SD; cm)	168.7 ± 6.2	168.9 ± 6.3	Ns	169.2 ± 6.1
BMI before pregnancy (mean ± SD)	22.2 ± 3.4	22.2 ± 3.3	Ns	21.9 ± 3.0
BMI at the delivery (mean ± SD)	27.4 ± 3.9	27.3 ± 3.7	Ns	27.0 ± 3.5
BMI increase (mean ± SD)	5.2 ± 1.8	5.1 ± 1.7	Ns	5.1 ± 1.7
Obstetrical characteristics				
Foetal weight (mean ± SD; g)	3357.1 ± 419.1	3381.6 ± 420.7	Ns	3362.0 ± 401.9
Length of the first stage (mean ± SD; hh:mm)	06:47 ± 3:59	06:53 ± 04:06	Ns	6:52 ± 04:07
Length of the second stage (mean ± SD; hh:mm)	00:44 ± 00:34	00:46 ± 00:35	Ns	00:43 ± 00:31
Elective caesarean section (*n*, %)	77 (3.3%)	50 (3.7%)	Ns	NA
Acute caesarean section (*n*, %)	469 (20.3%)	297 (22.2%)	Ns	NA
Forceps delivery (*n*, %)	37 (1.6%)	23 (1.7%)	Ns	23 (2.3%)
Vacuum extraction (*n*, %)	4 (0.2%)	3 (0.2%)	Ns	3 (0.3%)
Labour induction (*n*, %)	454 (19.6%)	312 (23.4%)	0.004	200 (20.3%)
Epidural analgesia (*n*, %)	541 (23.4%)	304 (22.8%)	Ns	147 (14.9%)
Oxytocin (*n*, %)	1545 (66.8%)	903 (67.6%)	Ns	692 (70.1%)
Use of analgesics other than epidural (*n*, %)	864 (37.4%)	525 (39.3%)	Ns	401 (40.6%)
Perineal rupture grade I (*n*, %)	228 (9.9%)	110 (8.2%)	Ns	110 (11.1%)
Perineal rupture grade II (*n*, %)	115 (5.0%)	77 (5.8%)	Ns	77 (7.8%)
Episiotomy (*n*, %)	1219 (52.7%)	703 (52.7%)	Ns	703 (71.2%)
Perineal rupture grade III (*n*, %)	37 (1.6%)	30 (2.2%)	Ns	30 (3.0%)

**Table 2:** Objective and subjective outcomes in *n* = 978 women in this study. The Oxford score was calculated as the average of right and left muscle strength

POP Q	Mean	SD; range
Aa	−1.6	0.6; −3 - +1
Ba	−1.6	0.6; −3 - +1
C	−5.8	1.5; −7 - +1
Ap	−1.5	0.6; −2 - +1
Bp	−1.5	0.7; −3 - +1
Pb	3.7	0.4; 2–7
Gh	3.8	0.4; 2–5
TVL	8.8	0.5; 6–10
Mean Oxford score	1.4	1.1; 0–5

Prolapse stage	N	%
Anterior stage I	0	–
Stage II	349	35.5.%
Central stage I	190	19.3%
Stage II	2	0.2%
Posterior stage I	0	–
Stage II	423	43.0%

Ultrasound findings (cm^2^)	Mean	SD; range
Urogenital hiatus on relaxation	22.8	4.2; 8.8–37.0
On valsalva	28.2	6.9; 8.22–49.7
During contraction	14.5	3.6; 6.6–30.5
Urethral gap	2.4	0.4; 1.6–4.7

Questioners	Median/mean	IQR/SD; range
ICIQ SF (*n* = 987)	1.9	4
ICIQ SF with UI (*n* = 314)	5.9	4
Amount of urine	2.0	0
UI frequency	1	0
UI visual analogue scale	2	2
PISQ 12	38.8	4.0; 13–47

### Levator muscles and degree of prolapse

In most women, the LAM evaluation was possible; in 13 cases, the LAM avulsion was evaluated together with the supervisor. Unilateral LAM avulsion was diagnosed 1 year after delivery in 173 (18.1%). In 89 (9.0%), this was bilateral. LAM avulsion was predominantly left (*n* = 109; 63%). Ballooning was present in 309 (31.3%) women, of which 165 (53.3%) were without LAM avulsion. The POP-Q, average Oxford score and subjective outcome are displayed in Supplementary Table 1. Stage II prolapse in at least one compartment was present in 562 (56.9%) of which stage II+ was present in 130 (23.1%).

### Urinary incontinence and urgency

**Fig. 1:**
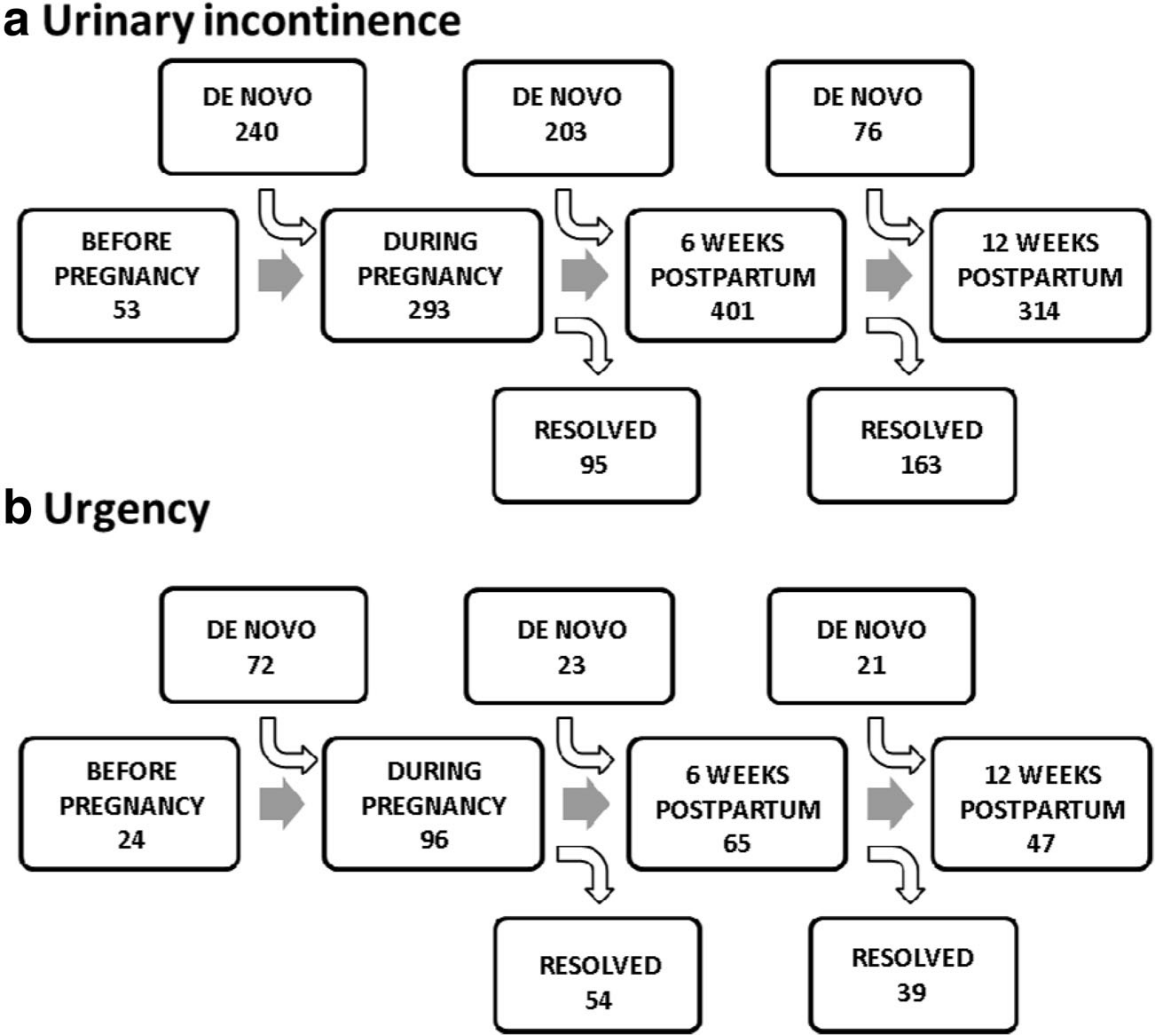
Development of urinary incontinence (A) and urgency (B)

Fifty-three (5.4%) women reported UI before pregnancy. That number increased six-fold during pregnancy to 29.7% of study participants (Fig. 1). Of those, in one-third (23.4%) this resolved within 6 weeks after birth, without reappearance within a year. However, after birth an additional 203 women who were previously not incontinent reported UI: this is nearly as many women as developed UI in pregnancy. As a result, 6 weeks postpartum, 40.6% (*n* = 401) were not dry. Later during that first year, UI resolved in 163 (− 40.6%), but 76 (+19.0%) women developed de novo incontinence. As a result, in our cohort, 31.8% of women still reported urinary incontinence after their first vaginal delivery. This population includes 66.0%(*n* = 35) of the women who reported UI before pregnancy, 41.7% (*n* = 100) of the women who developed UI during pregnancy, and 50% (*n* = 103) of the women who developed UI only during the postpartum period. The type of incontinence as picked up by the ICIQ SF (Domain 6) in these 314 women was during coughing and physical exercises in 61% before getting to the toilet in 20% and on mixed occasions in 19%.

Twenty-four (2.4%) women reported urgency before pregnancy. Their problem persisted in pregnancy, and their number increased four-fold (*n* = 96; 9.7%) (Fig. 1). In half of them, the issue resolved in the postpartum period, but 23 women developed de novo urgency after vaginal birth. By 1 year, the problem disappeared in half of the women. By 1 year, 4.8% of women who delivered vaginally reported urgency, of which half developed this as a new problem.

### Anal function

No women reported AI before or during pregnancy. Other dysfunctions were not quantified. At six weeks after birth, 1.6% of women reported faecal urgency (*n* = 2), flatus (*n* = 6) or stool (*n* = 8) incontinence. When adding the other forms of anorectal dysfunction, 8.4% of women reported bother at 6 weeks (*n* = 83). Three-quarters of these dysfunctions resolved by 1 year. AI persisted in only one woman, who sustained perineal rupture grade III. Eight additional women reported first AI between 6 weeks and 1 year after birth (6 urgencies, 2 flatus/stool incontinence). The number of women reporting de novo dyschezia was, however, three times higher (*n* = 24; 2.4%), which is half as many women reporting urgency incontinence.

### Sexual function

One year postpartum, 961(97.5%) women were sexually active, of which 169 (17.1%) reported dyspareunia. Women breastfeeding at 1 year (421, 42.6%) were more likely (OR: 1.449; 95%CI: 1.039–2.019, *p* = 0.033) to report dyspareunia.

### Uni- and multivariate analysis

We performed five sets of analysis for PFDs and LAM trauma at 12 months, the presence of urinary incontinence, pelvic organ prolapse stage II+, LAM avulsion and LAM ballooning (Tables 3, 4). Analysis of anal incontinence was not performed because of its low occurrence.

**Table 3:** Uni- and multivariate regression analysis for the presence of urinary incontinence (UI) and pelvic organ prolapse stage II+ (POP II+). Results for variables with *p* ˃ 0.250 in univariate analysis are not shown

	Univariate analysis		Multivariate analysis	
*n* = 987	OR (95% CI)	*p*	OR (95% CI)	*p*
UI				
Age (per additional year of age)	1.084 (1.041–1.129)	0.0001	1.088 (1.044–1.134)	0.0001
Height (per additional cm)	0.978 (0.957–1.000)	0.051	0.976 (0.837–0.988)	0.030
BMI before pregnancy	1.071 (1.026–1.119)	0.002	1.081 (1.035–1.130)	0.001
BMI at delivery	1.029 (0.991–1.068)	0.130		Excl.
BMI increase	0.899 (0.8285–0.976)	0.010	0.902 (0.828–0.979)	0.014
POP II+				
Age	1.081 (1.023–1.143)	0.006	1.082 (1.024–1.144)	0.005
Duration of the first stage (per additional minute)	0.999 (0.998–1.000)	0.035	0.999 (0.098–1.000)	0.032
Duration of the second stage (per additional minute)	0.996 (0.990–1.002)	0.238		Excl.
Foetal weight (per additional gram)	1.000 (1.000–1.001)	0.144		Excl.
Use of analgesics other than epidural	0.792 (0.537–1.168)	0.247		Excl.

**Table 4:** Uni- and multivariate regression analysis for presence of any levator ani avulsion (LAM avulsion) and ballooning without LAM avulsion. Results for variables with *p* ˃ 0.250 in univariate analysis are not shown

	Univariate analysis		Multivariate analysis	
*n* = 987	OR (95%CI)	*p*	OR (95% CI)	*p*
LAM avulsion				
Age (per additional year of age)	1.038 (0.995–1.082)	0.081		Excl
Initial BMI	0.964 (0.917–1.014)	0.152		Excl
Delivery BMI	0.964 (0.924–1.006)	0.093	0.952 (0.910–0.996)	0.032
Duration of the second stage (per additional minute)	1.006 (1.002–1.011)	0.007	1.005 (1.000–1.009)	0.044
Foetal weight (per additional gram)	1.001 (1.000–1.001)	0.003	1.001 (1.000–1.001)	0.007
Forceps delivery	4.841 (2.006–11.679)	0.0001	3.217 (1.538–8.223)	0.015
Epidural analgesia	0.633 (0.410–0.978)	0.042	0.576 (0.370–0.898)	0.015
Episiotomy	1.271 (0.920–1.755)	0.150		Excl
Perineal rupture grade I	0.466 (0.272–0.798)	0.004	0.495 (0.286–0.854)	0.012
Ballooning without LAM avulsion				
Age (per additional year of age)	1.061 (1.010–1.115)	0.019	1.075 (1.022–1.131)	0.005
Initial BMI	1.059 (1.005–1.116)	0.015	1.066 (1.010–1.125)	0.019
Delivery BMI	1.033 (0.987–1.082)	0.162		Excl
Duration of the second stage (per additional minute)	0.993 (0.987–0.999)	0.017	0.992 (0.986–0.998)	0.008
Perineal rupture grade III	0.347 (0.082–1.475)	0.208		Excl
Epidural analgesia	1.564 (1.016–2.409)	0.053	1.644 (1.059–2.551)	0.027
Episiotomy	0.671 (0.470–0.956)	0.030		Excl
Perineal rupture grade I	1.739 (1.084–2.790)	0.029	1.788 (1.103–2.899)	0.018
Use of analgesics other than epidural	0.711 (0.498–1.016)	0.065		Excl

Urinary incontinence was 1.6 more likely in women with LAM avulsion (95% CI: 1.175–2.127, *p* = 0.003). POP stage II+ was more likely in women with LAM avulsion (OR 2.588, 95% CI: 1.764–3.797, *p* = 0.0001) and with ballooning (OR 2.144, 95% CI: 1.396–3.293, *p* = 0.0001).

## Discussion

### Main findings

One year postpartum the most common PFD was urinary incontinence, reported by every third woman. Risk factors for its development were maternal age, BMI before pregnancy and its increase during pregnancy. The main risk factor for POPII+ was maternal age. LAM injuries were present in 43% of women. Muscle avulsion was 3.2 more likely in women who had forceps-assisted birth. Surprisingly, there was an opposite effect of EDA and perineal rupture grade I on the LAM avulsion and ballooning.

The study cohort is representative of the population of nulliparous women delivering at the Institute for most parameters. In the study cohort, 70% of women were administered oxytocin during labour, which is a result of the active labour management, which requires a progression of at least 1 cm/h [16]. There were no more details on administration; therefore, we were not able to discriminate between a short and extended application. However, higher maternal age (> 35 years) was shown to increase the likelihood of oxytocin administration two-fold compared with younger women (< 19 years) [19]. Also strikingly high was the number of episiotomies (71%), which surpasses the nationally reported rates [20]. We could not identify the possible reasons. There was a relatively low rate of EDA (14%), forceps/VEX deliveries (2.6%) and third-degree perineal ruptures (3.0%), all of which correspond to the long-term institutional and national data [21].

### Interpretation

Pregnancy and not only vaginal birth severely interferes with pelvic floor function. Every fourth woman reported stress UI only due to the pregnancy, which persisted in many of them until 1 year postpartum. After birth, more than one-third of asymptomatic women developed UI continuing in two-thirds of them beyond 1 year. Discriminating between the effects of the pregnancy itself and the impact of birth is not possible here.

Further evaluation of women with caesarean section may support the findings of others who have shown an increased risk of UI compared with nulliparas [22]. Similar to other studies, the age at first birth was confirmed to be critical for the development of symptomatic UI [23]. However, its effect attenuates with the actual age and disappears after 50 years of age [24].

Regression of UI during the observational period was probably also affected by promoted physiotherapy, but no more details were collected so we were not able to draw more conclusions. However, recent literature supports PFT as an appropriate treatment for UI even though it may not have a long-lasting effect [25].

For POP evaluation, we chose the more strict criteria because we lacked the subjectively reported outcome [13, 26]. Chances for POP(II+) increased by 8% for each additional year of age. Also, women who had LAM avulsion or ballooning were more likely to have POPII+. We did not include this factor in the analysis since it only develops as a result of birth. There is some evidence that mediolateral episiotomy protects against, whereas spontaneous perineal lacerations promote POP development [24]. We did not confirm this observation concerning POP because of either the high episiotomy rate or short observational period.

The underlying conditions for PFD development are direct and indirect injuries to all parts of the pelvic floor. We only investigated LAM. Avulsion was present in almost every third woman and was three-fold more likely in women with forceps delivery, which is similar to the current literature (OR 1.6–4.40) [5]. Unlike others, the unilateral avulsion was predominantly on the left side [27, 28]. Ballooning was often present in women with LAM avulsion, but in the regression, we only include ballooning in those without avulsion. Ballooning is a result of the muscular over-distension, micro-traumatization, pudendal neuropathy and resulting healing [24]. LAM avulsion and ballooning shared three risk factors (EDA, perineal rupture grade I and the second stage) but with opposite effects. The likelihood of LAM avulsion was halved in women who had EDA and who sustained grade I perineal rupture. On the other hand, the muscle is 1.6-fold and 1.8-fold more likely to become overstretched, respectively. The EDA effect could be explained by the resulting muscle relaxation, which becomes less likely to tear but more likely to be overstretched. Our observation could be supported by studies showing a protective effect of EDA on third- and fourth-degree perineal ruptures [29]. EDA also prolonged the second stage of labour and was previously linked with an increased risk of UI [7].

The role of grade I perineal rupture could be explained by compliant tissues allowing sufficient adaptation during crowning. Therefore, LAM avulsion is less likely, whereas overstretching (ballooning) is more likely. Since no prevention of perineal injury has been identified, the explanation could be an individual’s physiognomy and intrinsic tissue characteristics such as its composition, compliance and elasticity [30]. These are partially inherited, affected by age, affected by internal diseases, etc. [3]. Indeed, others have also identified age as a risk factor increasing the likelihood of LAM avulsion by 8–10% for each additional year [8].

### Limitations

The major limitations are the missing questionnaire on POP, missing information on anal incontinence and that they were not asked about typical symptoms (vaginal bulge, etc.). To overcome this, in the analysis we only considered POPII+, which includes descent to the level of the hymen or beyond [31]. We also did not prospectively collect subjective complaints on PFD before and during the pregnancy; therefore, these data may be subject to recall bias. We considered the collection of these data at admission inappropriate and not feasible. It would be beneficial also to obtain 3D scans of the anal sphincter to identify occult anal sphincter ruptures. We also did not use any standardized test to categorize the type of UI and relied on subjective reports. Another limitation is the 63% drop-out rate, which may have resulted in more women with PFD being included in the study group. During the follow-up, women were contacted and offered another appointment, but many of them did not have time or were not willing to continue the study.

### Strengths

The strengths comprised a large unselected cohort included in the follow-up and its prospective design. This was possible because of a high volume centre (> 5000 deliveries/year). This cohort was homogeneous since it only includes Caucasian women and therefore very well represents the local population, but the results may not be generalisable to other groups, e.g., non-Caucasians. For the future, we can follow them beyond the end of this study and collect data on subsequent deliveries and PFDs during their later lives. An additional benefit was an increase of general awareness of PFD among the population of women invited to the study and their peers who often contacted the urogynecological office to consult or treat their problems.

## Conclusion

It seems that we will never be able to prevent the development of PFD since part of their origin lies in the pregnancy and ageing. We should try to minimize the number of forceps extraction procedures, but not at the expense of foetal safety. Moreover, clinicians should inform women about their risks of PFD development as a result of pregnancy and delivery; however, it should not serve as a general excuse for performing a caesarean section. During the postpartum visit, midwives and gynaecologists should ask women about PFD, and if needed they should recommend preventive approaches (PFT, weight reduction, etc.).

To a certain extent, we have contributed to the discussion on risk factors related to PFD. Maternal age and weight were again identified as essential factors in the development of UI and POP. It seems that EDA may have a protective effect against LAM avulsion, but at the same time, it may contribute to micro-traumatization and the development of ballooning. To make a stronger conclusion on EDA impact, we would need more research. The effect of perineal rupture grade I may represent a link between the intrinsic properties of the tissues that allow some primiparous women to deliver with only minor injuries but predispose them to overstretched tissue.

## Electronic supplementary material

ESM 1 (DOCX 8 kb)
