# Supplementary material for: The effect of the first vaginal birth on pelvic floor anatomy and dysfunction
Source: Int Urogynecol J. 2019 Jul 20;30(10):1689–96. doi: 10.1007/s00192-019-04044-2 (PMC6795623; doi:10.1007/s00192-019-04044-2)
Supplement: Supplementary file 1 — (DOCX 8 kb) [file 192_2019_4044_MOESM1_ESM.docx]

Supplementary material:

Suppl. mat.1. The Interclass correlation (ICC), 95% confidential coefficient and the Kappa coefficient for the measure of agreement for the initial 55 cases evaluated by all observers.

| parametr | ICC | 95%CI |
| --- | --- | --- |
| Urogenital hiatus on relaxation | 0.68 | 0.52-0.80 |
| Urogenital hiatus during contraction | 0.78 | 0.65-0.87 |
| Urogenital hiatus on Valsalva | 0.77 | 0.65-0.86 |
| Urethral gap (right) | 0.69 | 0.53-0.81 |
| Urethral gap (left) | 0.64 | 0.46-0.77 |
|  | kappa |  |
| MLA avulsion | 0,827 |  |
